# The relationship between uric acid and total femur bone mineral density in hypertensive and non-hypertensive populations

**DOI:** 10.3389/fendo.2022.1022031

**Published:** 2022-12-19

**Authors:** Yingjie Su, Ning Ding, Yang Zhou, Guifang Yang, Xiangping Chai

**Affiliations:** ^1^ Department of Emergency Medicine, The Second Xiangya Hospital, Central South University, Changsha, Hunan, China; ^2^ Emergency Medicine and Difficult Diseases Institute, Central South University, Changsha, Hunan, China; ^3^ Trauma Center, Changsha, Hunan, China

**Keywords:** uric acid, bone mineral density, hypertension, NHANES, epidemiology

## Abstract

**Objective:**

This study aimed to explore the association between uric acid (UA) and total femur bone mineral density (BMD) in hypertensive and non-hypertensive groups.

**Methods:**

We conducted a cross-sectional study of 13,108 participants in the NHANES database, including 4,679 hypertensive and 8,429 non-hypertensive subjects. A weighted multiple linear regression analysis was conducted to explore the association between UA and total femur BMD.

**Results:**

In the hypertensive group, the relationship between UA and total femur BMD was positive [β, 3.02 (95% CI, -0.44 to 6.48), p = 0.0962). In the non-hypertensive group, the association was significantly positive [β, 5.64 (95% CI, 2.06–9.22), p = 0.0038]. In gender-stratified analysis, UA was analyzed as a continuous variable and a categorical variable (quartile). The significantly positive association was present in both the hypertensive male group [β, 5.10 (95% CI, 0.98–9.21), p for trend = 0.0042] and non-hypertensive male group [β, 10.63 (95% CI, 6.32–14.94), p for trend = 0.0001]. A smooth curve fitting showed that in the hypertensive male group, the relationship between UA and total femur BMD was an inverted U-shaped curve. In the hypertensive female group, the relationship was basically negative. In the non-hypertensive population, the relationship between UA and total femur BMD was an inverted U curve in both men and women.

**Conclusion:**

In the hypertensive male group, the association between UA and total femur BMD was an inverted U-shaped curve. As to women, the relationship was basically negative. In the non-hypertensive group, the association between UA and total femur BMD was an inverted U-shaped curve in different genders.

## Introduction

Hyperuricemia has been proven to be associated with a variety of clinical diseases, such as hypertension, heart failure, insulin resistance, diabetes, osteoporosis, and chronic renal disease (1–7). Previous studies have shown that both blood pressure and hypertension tend to increase with elevated uric acid (UA) levels (8–10). According to NHANES 2015–2016 data, in the US population, the prevalence of hyperuricemia in women was 20.2%, and that in women was 20.0% (11). In recent years, the relationship between UA and bone mineral density (BMD) or osteoporosis has also been discussed a lot. A meta-analysis of 55,859 subjects showed that UA levels were positively associated with BMD and negatively associated with new fractures (12). A meta-analysis of five prospective studies including 29,110 participants showed that UA levels were inversely associated with fracture risk, with an overall hazard ratio of 0.79 (95% CI, 0.69–0.89) in the highest group compared to the lowest group after tertiary grouping of UA levels (13). High oxidative stress levels and weak antioxidant capacity may be the underlying mechanisms leading to osteoporosis (14, 15). The antioxidant effect of UA may be used to explain the protective effect on BMD (16).

Inversely, few studies have investigated the relationship between UA and BMD in hypertensive and non-hypertensive populations. In this population, especially the hypertensive population, whether there is a correlation between low UA levels and decreased BMD is a question that deserves the attention of clinicians. As consequence, a cross-sectional study of 13,108 participants was used to investigate the relationship between UA and total femur BMD. The data on these participants were derived from the NHANES database.

## Methods

### Study population

**Figure 1:**
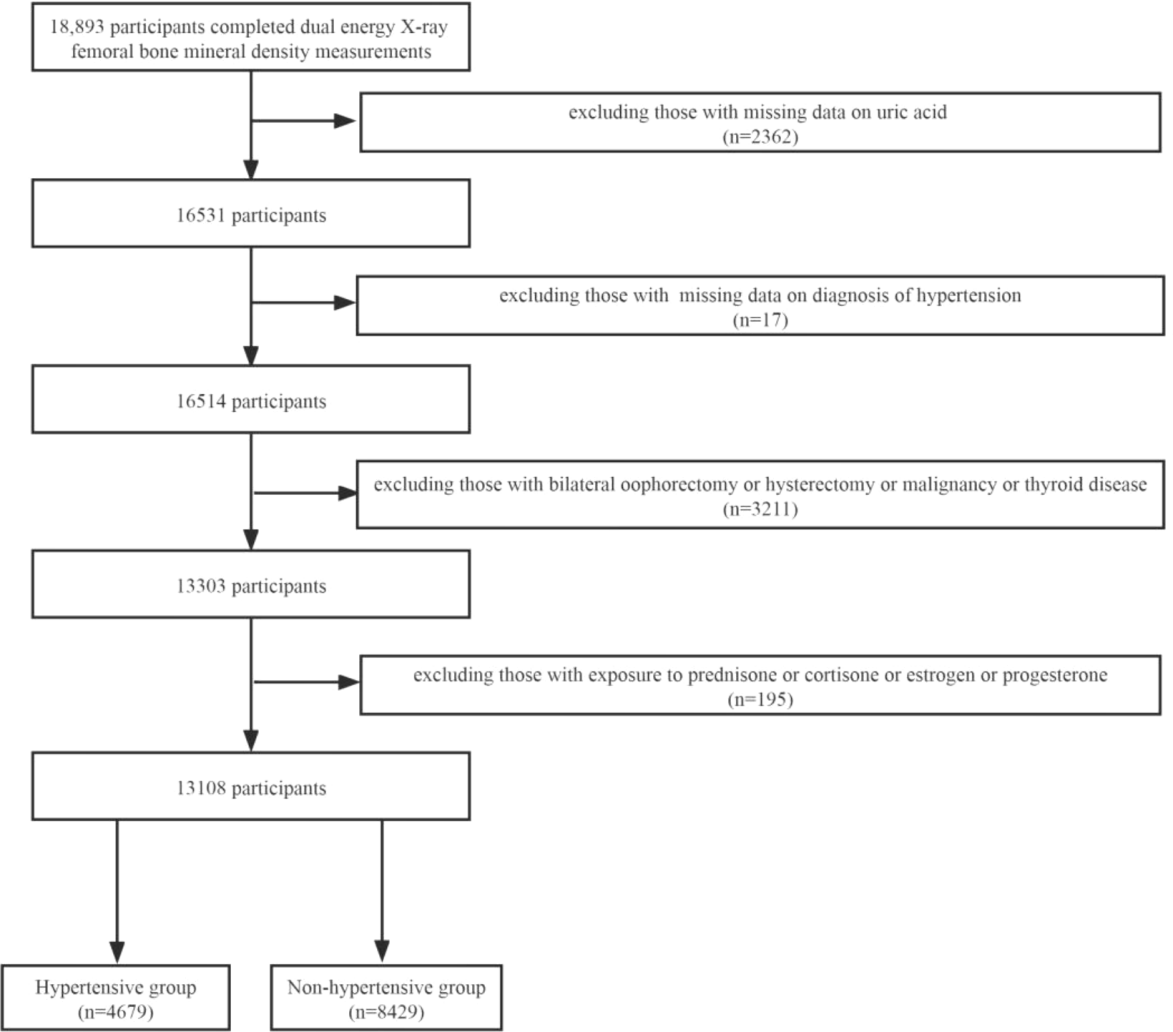
Flowchart of the study design and participants excluded from the study.

NHANES was a research program to investigate the health and nutritional status of the US population. It was counted on a 2-year cycle. A certain number of people are included in each cycle, and their sociology, lifestyle, diet, disease, blood testing, and other relevant indicators were collected. In our research, we collected data from four cycles of 2007–2008, 2009–2010, 2013–2014, and 2017–2018 for analysis (2011–2012, 2015–2016 without femoral BMD examinations). A total of 18,893 subjects completed femoral BMD scans during these four cycles, and we excluded 5,785 participants for the following reasons: 1) missing UA data (n = 2362); 2) missing data on the diagnosis of hypertension; 3) bilateral oophorectomy or hysterectomy or malignancy or thyroid disease (n = 3211); and 4) taking prednisone or cortisone or estrogen or progesterone. Finally, 13,108 participants were included in the study, including 4,679 in the hypertensive group and 8,429 in the non-hypertensive group (**Figure 1**).

### Bone mineral density and hypertension

In our study, total femur BMD was obtained by measuring the femur by dual energy X-ray absorptiometry (DXA). DXA was widely used in the measurement of BMD and has the advantages of fast speed, convenience, and low radiation (17). Three Hologic QDR-4500A fan-beam densitometers were used for total femur BMD scans, which were performed by trained and certified radiologic technicians. Longitudinal monitoring of the instrument by weekly scanning of the Hologic Femur Phantom was conducted according to the manufacturer’s requirements. Participants meeting one of the following four criteria will be defined as having hypertension: 1) systolic blood pressure (SBP) ≥140 mmHg, 2) diastolic blood pressure (DBP) ≥90 mmHg, 3) having been told by a doctor to have high blood pressure, and 4) taking antihypertensive medication. The SBP and DBP were obtained by the following method: All participants had their blood pressure measured by trained and certified personnel after a 5-min rest in a seated position. Three consecutive blood pressure readings were obtained by auscultation, and if blood pressure measurement was not completed successfully, a fourth measurement was taken. SBP and DBP are averages of all available measurements.

### Sociodemographic and lifestyle factors

We included three demographic variables: gender, age, and race. Lifestyle included smoking status, alcohol consumption, and total physical activity (TPA). Smoking status was classified according to multiple questions in the smoking questionnaire: current smoking, former smoking, non-smoking, and unrecorded. Lifetime smoking of less than 100 cigarettes was defined as non-smoking. Alcohol consumption was classified as drinking, non-drinking, and unrecorded according to questions on the drinking questionnaire. Among them, drinking more than or equal to 12 times per year was defined as drinking. TPA data were based on the Global Physical Activity Questionnaire, which includes three types of physical activity: recreational, occupational, and transportation, and were divided into vigorous and moderate categories according to the intensity of each physical activity. TPA is the sum of the three types of physical activity (multiplied by two if the physical activity was vigorous) (18, 19). According to the 2018 Physical Activity Guidelines for Americans, subjects were defined as active participants if their TPA minutes were ≥150 min (20).

### Metabolic factors, clinical laboratory indicators, and dietary factors

Metabolic factors and clinical laboratory indicators include diabetes, body mass index (BMI), total cholesterol, glomerular filtration rate (GFR), serum vitamin D2 + D3, and UA. Participants meeting one of the following four criteria will be defined as having diabetes:1) taking hypoglycemic agent or insulin, 2) fasting blood sugar ≥126 mg/dl, 3) 2-h Oral Glucose Tolerance Test blood sugar ≥200 mg/dl, and 4) glycohemoglobin ≥6.5%. BMI was calculated as weight in kilograms divided by height in meters squared. The Modification of Diet in Renal Disease (MDRD) equation was used to calculate GFR: 186 × SC^−1.154^ × Age^−0.203 ^× (0.742 if female) (21). Dietary variables incorporate daily total protein and total calcium intake. Two 24-h dietary recall recordings were conducted by trained dietary interviewers. Total protein and total calcium intakes were the mean of protein and calcium intakes recorded from two dietary recalls. In the hypertensive group, whether antihypertensive treatment was administered was also included in the data analysis.

### Statistical analysis

Appropriate weights, stratification, and clustering were used in the data analysis in order for the findings to be representative of the US population. In hypertensive and non-hypertensive groups, the correlation between UA and total femur BMD was determined by the Pearson correlation test. We use a weighted mean [95% confidence intervals (CI)] representation for continuous variables and weighted percentage (95% CI) representation for categorical variables. Continuous and categorical clinical characteristics were compared using survey-weighted linear regression and survey-weighted chi-square tests, respectively. The independent relationship between UA and total femur BMD in the hypertensive and non-hypertensive groups was conducted by applying the multiple linear regression model. At the same time, the linear trend test was performed after grouping the UA into quartiles. To discover the nonlinear relationship between UA and total femur BMD, we performed smooth curve fittings. All variables were incorporated into the model to adjust for potential confounding, and analyses were stratified by gender. Data analysis was performed using the statistical packages R (http://www.R-project.org) and EmpowerStats (http://www.empowerstats.com). Differences were considered statistically significant when p-value <0.05.

## Results

### Subjects characteristics

The baseline characteristics of participants were illuminated in **Table 1**. In the hypertensive group (n = 4679), there was a significantly positive correlation between UA and total femur BMD (r = 0.2081, p < 0.0001). The ratios of men and women were 59.62% and 40.38%, respectively; 66.56% were non-Hispanic white. The proportions of smoking, drinking, active participants, and diabetes were 18.70%, 49.21%, 58.44%, and 24.38% respectively; 11.33% people have a GFR <60 ml/min/1.73 m^2^. The mean value for age, total cholesterol, BMI, serum vitamin D2 + D3, protein intake, calcium intake, total femur BMD, and UA were 56.54 years, 5.09 mmol/L, 29.93 kg/m^2^, 68.95 nmol/L, 82.52 g/day, 938.74 mg/day, 976.56 mg/cm^2^, and 5.83 mg/dl, respectively; 59.12% of the population received antihypertensive therapy. In the non-hypertensive group (n = 8429), there was a significantly positive correlation between UA and total femur BMD (r = 0.2735, p < 0.0001). The ratios of men and women were 54.00% and 46.00%, respectively; 65.48% were non-Hispanic white. The proportions of smoking, drinking, active participants, and diabetes were 18.56%, 47.89%, 72.72%, and 5.82% respectively; 1.74% people have a GFR <60 ml/min/1.73 m^2^. The mean value for age, total cholesterol, BMI, serum vitamin D2 + D3, protein intake, calcium intake, total femur BMD, and UA were 38.95 years, 4.89 mmol/L, 26.60 kg/m^2^, 68.87 nmol/L, 84.43 g/day, 1010.29 mg/day, 981.54 mg/cm^2^, and 5.25 mg/dl, respectively.

**Table 1:** Description of participants based on the presence or absence of hypertension.

	Hypertension(yes)	Hypertension(no)	p-Value
	N = 4679	N = 8429
Gender			<0.0001
Male	59.62 (57.99,61.23)	54.00 (52.74,55.26)	
Female	40.38 (38.77,42.01)	46.00 (44.74,47.26)	
Age(years)	56.54 (56.02,57.05)	38.95 (38.34,39.55)	<0.0001
Race			<0.0001
Mexican American	6.73 (5.12,8.81)	10.87 (8.58,13.68)	
Other Hispanic	5.13 (4.03,6.50)	6.27 (4.83,8.10)	
Non-Hispanic White	66.56 (62.11,70.73)	65.48 (61.35,69.38)	
Non-Hispanic Black	13.68 (11.41,16.32)	9.67 (8.41,11.10)	
Other Race	7.90 (6.64,9.37)	7.72 (6.42,9.24)	
Smoking status			<0.0001
Current-smoking	18.70 (16.91,20.64)	18.56 (17.19,20.00)	
Former smoking	29.41 (27.62,31.27)	17.94 (16.52,19.46)	
Non-smoking	51.19 (48.67,53.71)	47.54 (45.14,49.95)	
Not recorded	0.70 (0.53,0.92)	15.96 (15.05,16.91)	
Alcohol consumption			<0.0001
No drinking	35.61 (33.13,38.17)	24.60 (23.16,26.10)	
Drinking	49.21 (46.73,51.69)	47.89 (46.05,49.73)	
Not recorded	15.18 (13.83,16.64)	27.51 (26.15,28.91)	
Total physical activity			<0.0001
Inactive participants	41.56 (39.52,43.62)	27.28 (25.84,28.78)	
Active participants	58.44 (56.38,60.48)	72.72 (71.22,74.16)	
Diabetes			<0.0001
No	75.62 (73.59,77.55)	94.18 (93.35,94.91)	
Yes	24.38 (22.45,26.41)	5.82 (5.09,6.65)	
GFR(ml/min/1.73 m^2^)			<0.0001
<60	11.33 (10.05,12.75)	1.74 (1.37,2.21)	
60-90	49.71 (47.52,51.91)	35.23 (33.54,36.97)	
≥90	38.96 (36.64,41.33)	63.03 (61.19,64.83)	
Total Cholesterol (mmol/L)	5.09 (5.04,5.13)	4.89 (4.86,4.93)	<0.0001
BMI (kg/m^2^)	29.93 (29.70,30.17)	26.60 (26.41,26.79)	<0.0001
Serum vitamin D2 + D3 (nmol/L)	68.95 (67.45,70.44)	68.87 (67.41,70.32)	0.9172
Protein intake (g/day)	82.52 (80.86,84.17)	84.43 (83.19,85.66)	0.0343
Calcium intake (mg/day)	938.74 (920.73,956.74)	1010.29 (988.48,1032.10)	<0.0001
Total femur BMD (mg/cm^2^)	976.56 (969.87,983.25)	981.54 (976.89,986.19)	0.2118
Uric acid (mg/dl)	5.83 (5.76,5.90)	5.25 (5.21,5.28)	<0.0001
Antihypertensive therapy			
Yes	59.12 (57.02,61.18)		
No	40.88 (38.82,42.98)		

GFR, Glomerular filtration rate; BMD, Bone mineral density; BMI, body mass index.

For continuous variables: survey-weighted mean (95% CI), p-value was by survey-weighted linear regression.

For categorical variables: survey-weighted percentage (95% CI), p-value was by survey-weighted chi-square test.

### Association between UA and total femur BMD

**Table 2** shows the results of weighted multiple linear regression analyses between UA and total femur BMD in different models. In model I, sociodemographic factors (gender, age, race) were adjusted. The significantly positive association can be found in the hypertensive [β, 11.06 (95% CI, 7.04–15.07)] and non-hypertensive group (β, 23.83 (95% CI, 20.25–27.40)]. In model II, lifestyle factors (smoking status, alcohol consumption, TPA) were adjusted and added into model I. The significantly positive association still presented in the hypertensive [β, 10.73 (95% CI, 6.69–14.77)] and non-hypertensive group [β, 22.68 (95% CI, 19.17–26.20)]. In model III, metabolic, clinical laboratory indicators, and dietary factors (BMI, GFR, total cholesterol, diabetes, serum vitamin D2 + D3, protein intake, calcium intake) were adjusted and added into model II. In the hypertensive group, whether or not to receive antihypertensive therapy was also included in the model to adjust. Although the positive association still presented [β, 3.02 (95% CI, -0.44 to 6.48)], the p-value was >0.05. In the non-hypertensive group, a significantly positive association still existed [β, 5.64 (95% CI, 2.06–9.22)].

**Table 2:** Result of multiple linear regression analysis between UA and total femur BMD in hypertensive and non-hypertensive group.

Model	Hypertensive group (β, 95% CI, p)	Non-hypertensive group (β, 95% CI, p)
Model I	11.06 (7.04, 15.07) <0.0001	23.83 (20.25, 27.40) <0.0001
Model II	10.73 (6.69, 14.77) <0.0001	22.68 (19.17, 26.20) <0.0001
Model III	3.02 (-0.44, 6.48) 0.0962	5.64 (2.06, 9.22) 0.0038

CI, confidence interval; UA, uric acid; BMD, bone mineral density.

For BMD: survey-weighted β (95% CI) p-value.

Model I was adjusted for: gender, age, and race.

Model II was adjusted for: smoking, alcohol consumption, and total physical activity in addition to model I.

Model III was adjusted for: body mass index, glomerular filtration rate, total cholesterol, diabetes, serum vitamin D2 + D3, protein intake, and calcium intake, in addition model II. Antihypertensive treatment variables were adjusted in the hypertensive group.

### Subgroup analysis stratified by gender

**Figure 2:**
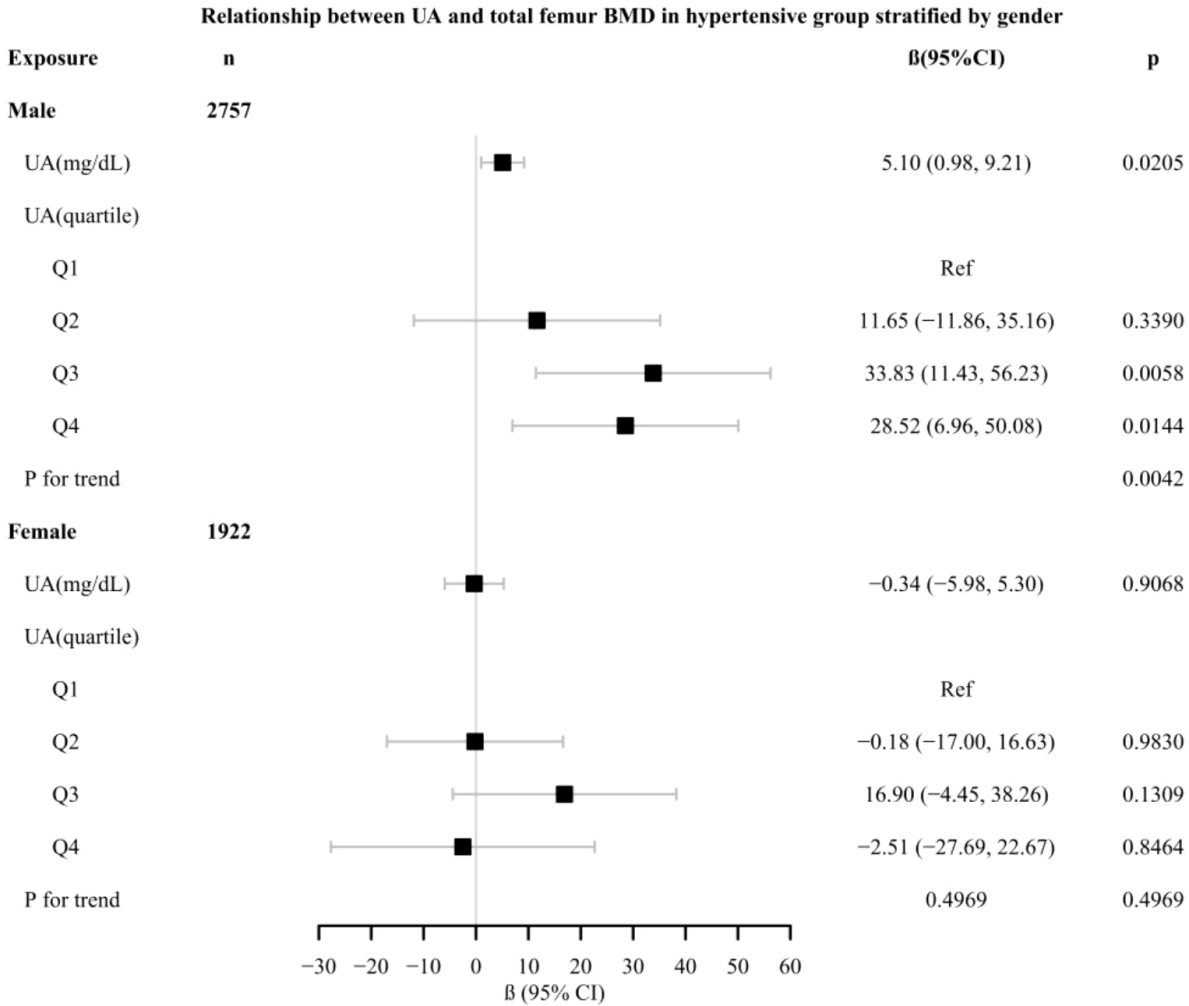
Relationship between UA and total femur BMD in the hypertensive group, stratified by gender. Adjusted for: race, age, alcohol consumption, smoke, total physical activity, body mass index, glomerular filtration rate, total cholesterol, diabetes, serum vitamin D2 + D3, protein intake, calcium intake, antihypertensive treatment.

**Figure 3:**
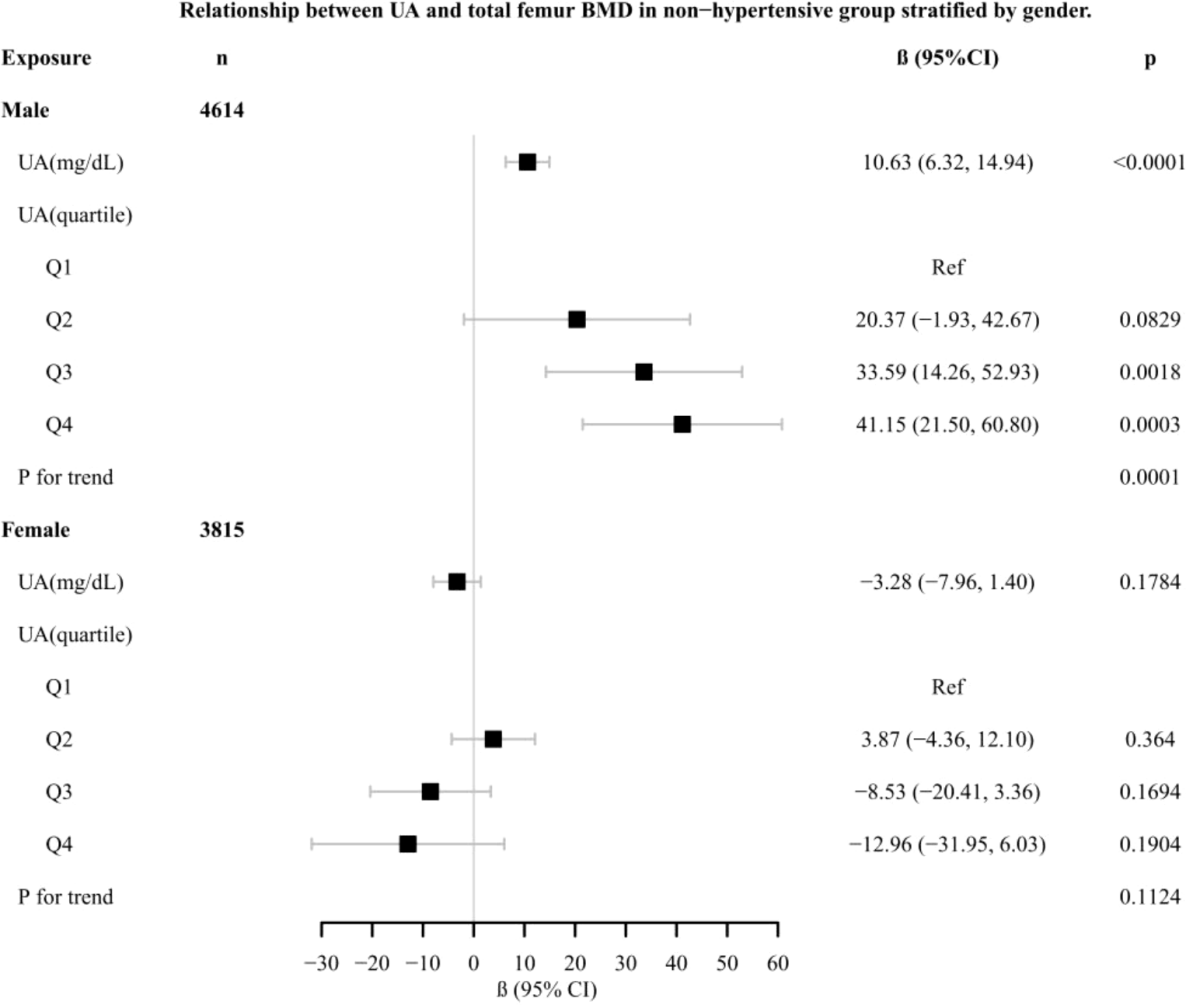
Relationship between UA and total femur BMD in the non-hypertensive group, stratified by gender. Adjusted for: race, age, alcohol consumption, smoke, total physical activity, body mass index, glomerular filtration rate, total cholesterol, diabetes, serum vitamin D2 + D3, protein intake, calcium intake.

**Figure 4:**
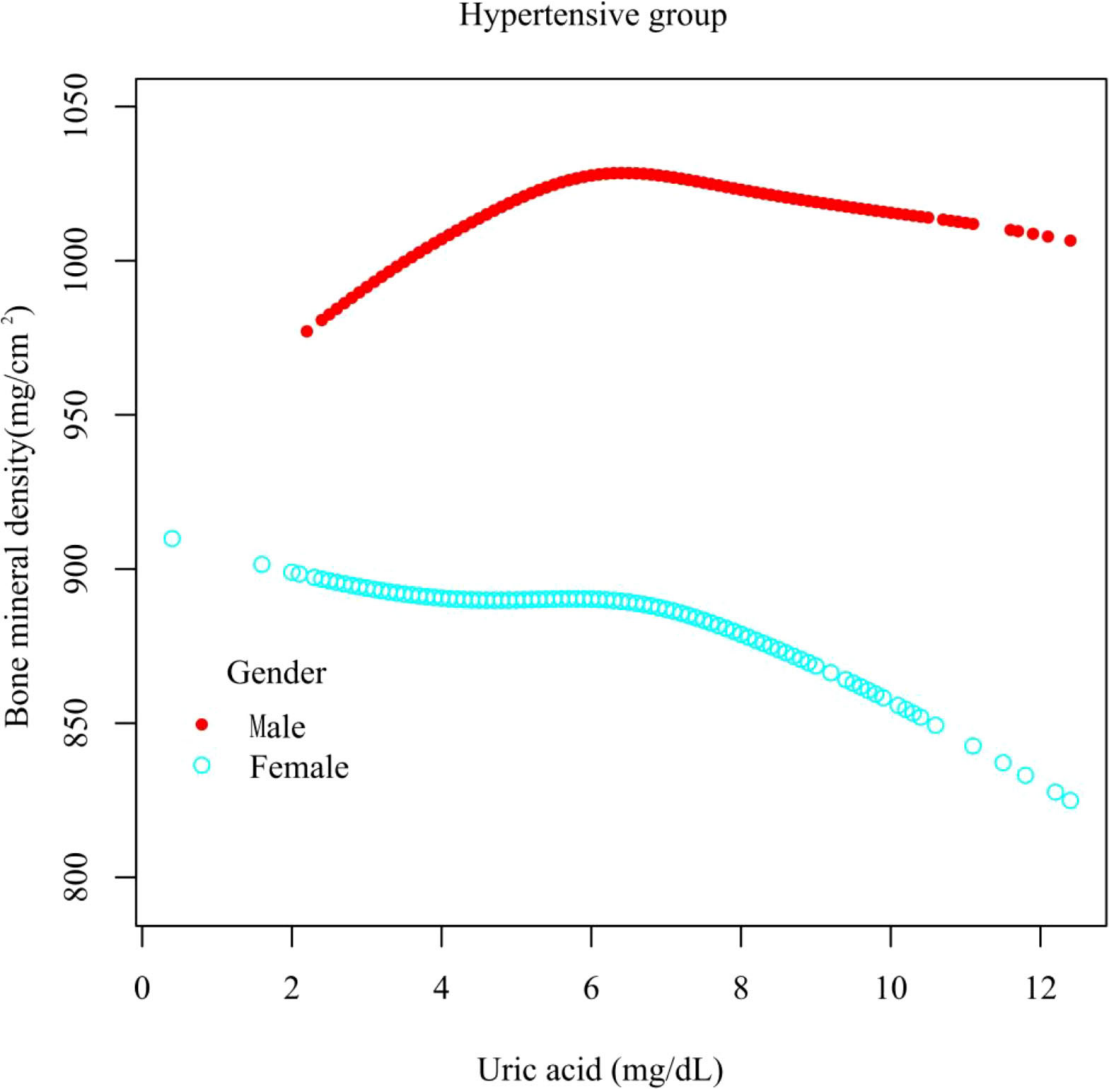
A smooth curve fitting for the relationship between UA and total femur BMD in the hypertensive group, stratified by gender. Adjusted for: race, age, alcohol consumption, smoke, total physical activity, body mass index, glomerular filtration rate, total cholesterol, diabetes, serum vitamin D2 + D3, protein intake, calcium intake, antihypertensive treatment.

**Figure 5:**
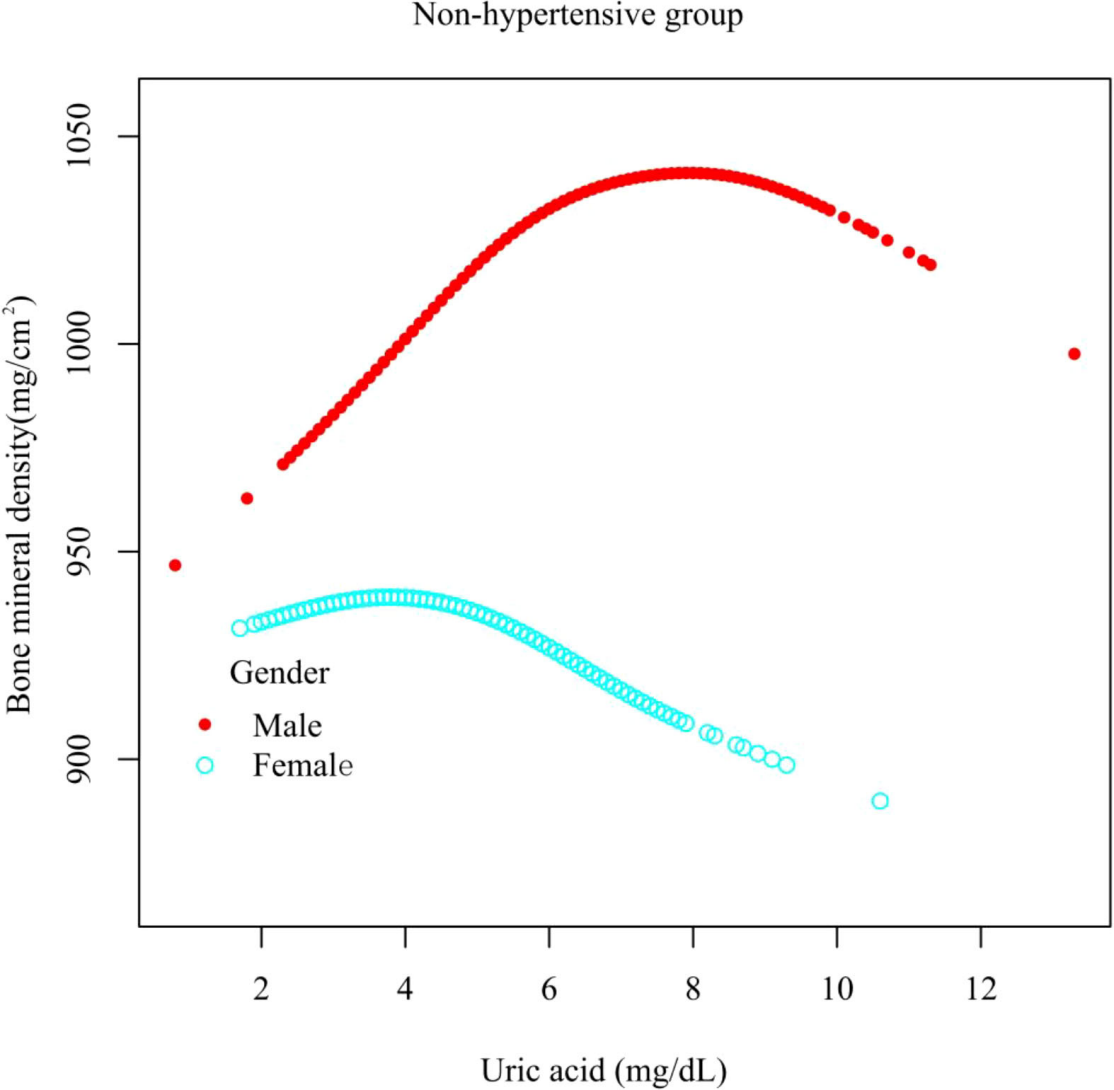
A smooth curve fitting for the relationship between UA and total femur BMD in the non-hypertensive group, stratified by gender. Adjusted for: race, age, alcohol consumption, smoke, total physical activity, body mass index, glomerular filtration rate, total cholesterol, diabetes, serum vitamin D2 + D3, protein intake, calcium intake.

In different subgroup analyses, we analyzed UA with continuous and categorical variables (quartile). In the hypertensive group (**Figure 2**), as to men, the relationship between UA and total femur BMD was significantly positive [β, 5.10 (95% CI, 0.98, 9.21)], and the trend test was significant (p = 0.0042). Paradoxically, this linear relationship and trend test were not significant in women. In the non-hypertensive group (**Figure 3**), the significantly positive association [β, 10.63 (95% CI, 6.32–14.94)] and trend test (p = 0.0001) still existed in men. In women, this linear relationship and trend test were also not significant. So to explore the nonlinear relationship, we performed a smooth curve fitting. In hypertensive male and female groups (**Figure 4**), the relationships were an inverted U-shaped curve and negative, respectively. In the non-hypertensive population (**Figure 5**), the relationship between UA and total femur BMD was an inverted U curve in both men and women.

## Discussion

In this study, the association of UA with total femur BMD in hypertensive and non-hypertensive populations was investigated in a large population (n = 13108). In the hypertensive male group, the association between UA and total femur BMD was an inverted U-shaped curve. As for the female, the relationship between the two was basically negative. In the non-hypertensive group, the association between UA and total femur BMD was an inverted U-shaped curve between different genders.

Many studies have reported the relationship between UA and BMD. However, there are some differences in the findings due to the different study groups. A cross-sectional study including 1705 men over 70 years of age suggested that men with serum UA levels above the group median had significantly higher BMD at all sites than men with UA levels below the median (22). Paradoxically, in a prospective cohort study of 1963 older men (age ≥65), high UA levels (≥6.88 mg/dl) were associated with an increased risk of hip fracture (23). In terms of women, in a study including 615 perimenopausal and postmenopausal Japanese women, higher UA levels were linearly associated with higher lumbar spine BMD (24). Inversely, in another recent study of 103,799 women, those with a history of gout had a 38% higher risk of hip fracture than those without a history of gout after 22 years of follow-up (25).

When we explored the relationship between UA and total femur BMD in both hypertensive and non-hypertensive male subgroups, the trend test was significant, and the smooth curve fitting showed an inverted U-shaped curve. The reason for this result was that, according to the quartiles of UA levels, women have lower UA levels than men, resulting in fewer men in the low group and a majority of men in the high group. Although the antioxidant effect of UA can prevent the loss of bone mass (16), studies have also shown that the higher the UA level, the lower the testosterone level (26, 27), and the lack of testosterone can lead to osteoporosis (28). This mechanism may be applied to explain the inverted U relationship between UA and total femur BMD in men. There were gender differences in the relationship between UA and BMD in the hypertensive group. Although the prevalence of chronic kidney disease (CKD) was higher among women than men, the rate of functional deterioration is faster in men than in women (29). Animal studies have shown that estrogen has a protective effect on the kidney (30) and that testosterone worsens kidney function (31), while kidney oxidative stress levels are lower in women (32). CKD causes hyperparathyroidism, increased UA levels, and decreased vitamin D activity, while UA decreased vitamin D levels and the parathyroid hormone decreased UA excretion. The interaction of multiple factors eventually leads to a decrease in bone mass (33) and gender differences in the hypertensive population. In hypertensive and non-hypertensive women, the relationship between UA and total femur BMD was negative and inverted U-shaped, respectively, which could be partly explained by the different levels of sex hormones. The mean ages of the hypertensive and non-hypertensive women in our study were 57.52 and 39.00 years, respectively; so most of the hypertensive women in our study were in menopause. After menopause, the lack of sex hormones can lead to decreased bone mass and osteoporosis (34). Nevertheless, the lack of estrogen and progesterone will reduce the function of the kidneys to excrete UA, resulting in an increase in the level of UA (35). Homoplastically, research has also shown that in postmenopausal women, higher levels of UA were associated with lower levels of vitamin D (36).

The significance of our study is to provide clinicians with some hints that appropriate UA-lowering therapy does not necessarily lead to bone loss in both hypertensive and non-hypertensive populations. Prospective studies are needed in the future to verify the effect of UA-lowering therapy on BMD in hypertensive and non-hypertensive populations. Of course, our study also has certain limitations. First, due to the limitations of cross-sectional study, the causal effect of UA on BMD has not been determined. Future prospective studies will be needed to clarify the relationship between them. Second, some variables in the study were based on self-report and may have subjective recall bias. Third, some participants in the hypertensive group were diagnosed based on the measurement of blood pressure multiple times in a short period of time, which may not necessarily reflect the state of blood pressure.

## Conclusions

In the hypertensive male group, the association between UA and total femur BMD was an inverted U-shaped curve. As to women, the relationship was basically negative. In the non-hypertensive group, the association between UA and total femur BMD was an inverted U-shaped curve between different genders.

## Data availability statement

The raw data supporting the conclusions of this article will be made available by the authors, without undue reservation.

## Ethics statement

The studies involving human participants were reviewed and approved by National Center for Health Statistics. The patients/participants provided their written informed consent to participate in this study. Written informed consent was obtained from the individual(s) for the publication of any potentially identifiable images or data included in this article.

## Author contributions

Conception and design: YS and ND; Administrative support: XC; Provision of study materials or patients: XC; Collection and assembly of data: YS, YZ, and GY; Data analysis and interpretation: YS and ND. All authors contributed to the article and approved the submitted version.
